# *Mocs1* (*Molybdenum cofactor synthesis 1*) may contribute to lifespan extension in Drosophila

**DOI:** 10.17912/micropub.biology.000517

**Published:** 2022-01-25

**Authors:** Eleanor I. Lamont, Michael Lee, David Burgdorf, Camille Ibsen, Jazmyne McQualter, Ryan Sarhan, Olivia Thompson, Sandra R Schulze

**Affiliations:** 1 Department of Biology, Western Washington University, Bellingham, WA, 98225, USA

## Abstract

While evaluating the effect on lifespan of decreased ribosomal protein (Rp) expression in Drosophila, we discovered a potential function in the same process for the *Molybdenum cofactor synthesis 1* (*Mocs1*) gene. We utilized the UAS-GAL4 inducible system, by crossing tissue-specific GAL4 drivers to the Harvard Drosophila Transgenic RNAi Project (TrIP) responder lines for Rp gene knockdown. We also employed a negative control that knocked down a gene unrelated to Drosophila (GAL4). Relative to the genetic background in which no driven transgenes were present, lifespan was significantly lengthened in females, both for Rp knockdown and the negative GAL4 control. We reasoned that the *Mocs1* gene, located immediately downstream of the integration site on the third chromosome where all the TrIP responders are targeted might be responsible for the lifespan effects observed, due to the potential for upregulation using the UAS-GAL4 system. We repeated the lifespan experiment using an enhancer trap in the same location as the TrIP transgenes, and found that lifespan was significantly lengthened in females that possessed both the driver and responder, relative to controls, implicating *Mocs1* in the biology of aging.

**Figure 1. Gene expression driven in the close vicinity of the Mocs1 gene appears to extend lifespan in Drosophila:**
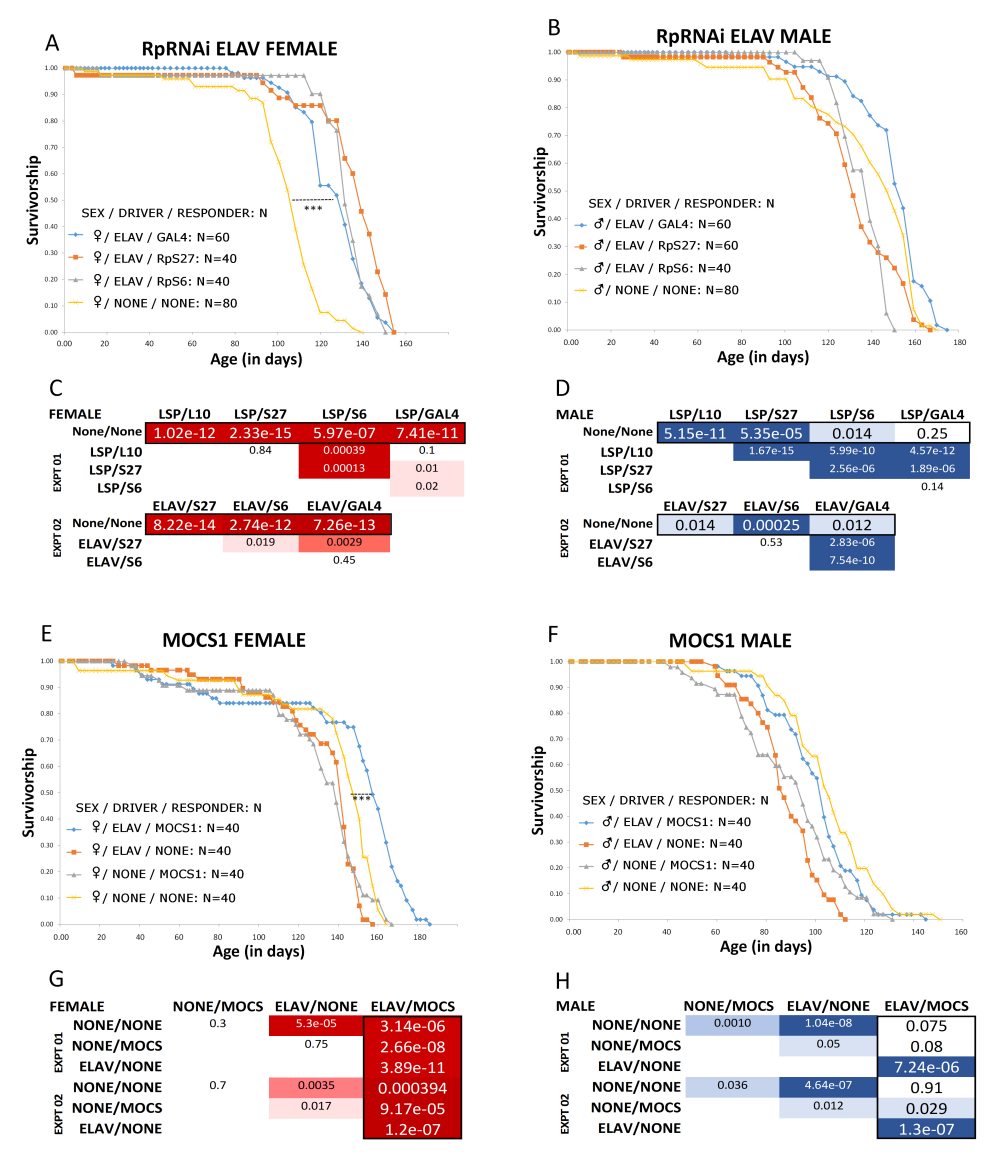
**Part A, B, E, F:** Representative Kaplan-Meier survival curves for ribosomal protein knockdown (RpRNAi: **A,B**) and MOCS1 putative upregulation (MOCS1: **C,D**). For the RpRNAi experiments (**A-D**), lifespan was extended a minimum of 21% and a maximum of 28% relative to the single background control, in females only, to a statistically significant degree (***), for all genotypes in which a responder was driven (both the RpRNAi responder, and the negative control GAL4 responder). The in-graph legend indicates fly genotype (the ELAV neuronal-specific GAL4 driver driving RNAi responders targeting RpS27, RpS6 or GAL4). The same analysis was performed using the LSP2 fat body GAL4 driver driving RNAi targeting RpS6, RpS27, RpL10 and GAL4 with highly similar results (statistical analysis for representative ELAV and LSP2 experiments shown in parts **C and D**). The genotypes using transgenic RNAi could not be isogenized (see description for details), so the negative (background) control consisted of progeny from a cross (in the same direction) between the genetic backgrounds of the driver and RNAi responder. For the MOCS1 experiments **(E-H),** lifespan was statistically significantly longer for the genotype in which both the ELAV driver and the MOCS1 enhancer trap were present. Lifespan extension was 6% minimum and 12% maximum relative to three negative controls, again, only in females. All the genotypes used in the MOCS1 experiments were isogenized for at least ten generations (see description for details). Parts **C, D, G, H** show tables of all pairwise log-rank tests for statistical significance, for representative experiments. All experiments (6 for RpRNAi and 2 for MOCS1) produced very similar results. Darkest hued cells represent the smallest P values (P<0.0001). Medium hued cells represent midrange low P values (0.001>P>0.01) light hued cells represent cut off for significance (0.01>P >0.05), and white cells represent data that are not significant. Relevant driver/enhancer combinations indicating lifespan extension are depicted in larger font and boxed in black in the tables. Total numbers for any given genotype for the RpRNAi experiments were 240-360 per sex (6 experiments total). Total numbers of flies for the MOCS1 experiments were 80 per sex (2 experiments total).

## Description

The experiment described in this paper resulted from a failed negative control in a study examining the effect of reducing ribosomal protein (Rp) gene expression on lifespan in Drosophila. Reduction in several different Rps has been shown to lengthen lifespan in a variety of model organisms (*C. elegans, S. cerevisiae, D. melanogaster* among others Steffen *et al.* 2008, Bell *et al*. 2009, Lindquist *et al.* 2011) presumably via impacts on Target of Rapamycin (TOR) signaling and possibly also mitochondrial function (Riera *et al.* 2016). As a means of knocking down Rp gene expression *in vivo*, we made use of the modularized miss-expression system consisting of GAL4 drivers (genetic strains of flies that express the yeast GAL4 transcription factor tissue-specifically) and responders (genetic strains of flies that possess GAL4 inducible transgenes expressing a gene of interest) (Rørth *et al.* 1998). When drivers and responders are crossed to each other, GAL4 induction of the gene of interest can be observed in the progeny. For the Rp experiment, we specifically employed transgenic RNAi responder lines from the Harvard Drosophila Transgenic RNAi Project (TrIP) where inducible transgenes expressing dsRNA against specific Rps were all integrated into a targeted locus on the third chromosome. This locus had been selected based on extensive expression analysis designed to minimize position effects that might shut down a transgene due to genomic location (Zirin *et al.* 2020). All the transgenes we used were located ~40 bp upstream of a gene called *Mocs1* (CG33048), which encodes a cofactor required by enzymes that utilize Molybdenum. In our RpRNAi lifespan experiment, we used maternally inherited neuronal and fat body GAL4 inducers (drivers) to knock down Rp gene expression (paternally inherited RNAi responders) in these specific (neuronal and fat body) tissues where the intersection between nutrient sensing and metabolism correlates with lifespan modulation (Shen *et al.* 2009, Hoffman *et al.* 2013, Fabian *et al.* 2021). The direction of the cross appeared to matter (i.e., which parent passed the driver or responder to the experimental offspring) as results were equivocal for the reciprocal cross in a pilot. As a first negative control for the RpRNAi experiment, we used progeny from a cross between the original strain the TrIP project used to *target* the RNAi transgenes (this contains the att-P2 “docking site” but no inducible GAL4 transgene) and the *w[1118]* isogenic strain which represented the driver background (see reagents). This combination served as a non-isogenic background control since the TrIP lines themselves could not be isogenized efficiently. (These lines are in a genetic background that makes it very difficult to follow the presence or absence of the transgenes by eye through multiple generations, necessitating a molecular approach that would effectively double the length of time in which to complete an already lengthy experiment – see methods below.) As a second negative control for the RpRNAi experiment, we used a TrIP line with a GAL4 inducible transgene expressing RNAi against GAL4 itself (this is a recommended control line from the TrIP project, see reagents; Zirin *et al.* 2020).

The surprising result was that all lines induced by GAL4, *including* the negative control driven GAL4 RNAi responder, showed a statistically significant lifespan extension in females relative to the non-induced genetic background control (**Figure 1A, B, C and D**). This experiment was repeated five times, with concealed genotypes using dLife software (Linford *et al.* 2013) to ensure data collection was unbiased and blind. A search of the literature regarding the effects of GAL4 induction and/or RNAi in Drosophila on lifespan were inconclusive, and then, only, for ubiquitous (as opposed to tissue-specific) drivers (Alic *et al.* 2012; Slade and Staveley 2015). Nevertheless, this UAS-GAL4 modularized system has been widely used for lifespan studies (Chavrous *et al*. 2001, Kapahi *et al.* 2004, Ruzzi *et al*. 2020). In addition, there is evidence for a QTL in the vicinity of the *Mocs1* locus that correlates with a longer lifespan in Drosophila (Tahoe *et al.* 2002 *Mocs1* called *low xanthine dehydrogenase* (*lxd)* at the time). We reasoned that driving expression in the *Mocs1* region might lead to its upregulation, which in turn may lengthen lifespan based on a hypothetical role for this gene’s product in regulating cellular protection in redox biochemistry (Zhang and Gladyshev, 2008). Thus, we repeated the lifespan experiment, this time using an enhancer trap (an inducible GAL4 transgene that will drive expression of genes near which it is located) in the same location (and correct orientation with respect to *Mocs1*) as the targeted RNAi transgenes from the TrIP resource (see reagents). Additionally, we isogenized the genetic background of both a single driver (ELAV) and the responder (enhancer trap) for ten generations, a critical procedure that controls for genetic background, which, as mentioned, is not feasible using the genotypes from the Harvard TrIP resource. We chose to focus on the ELAV pan-neuronal driver because *Mocs1* appears to be expressed predominantly in the nervous system (Schauer *et al*. 2013, Brown *et al.* 2014). The data are shown in **Figure 1: E, F, G, and H.** While the inferred position effect induction of *MocsI* was not directly measured by RTQPCR (see proposed future work below), we did observe a statistically significant lifespan extension in the experimental group (Drosophila bearing both the MOCS1 enhancer trap and the ELAV driver), albeit, and again, only in females.

How would putative upregulation of *Mocs1* contribute to lifespan extension? Molybdenum is a transition metal utilized across prokaryote and eukaryote taxa in metabolism, but it typically requires an organic compound (a pyranopterin) for enzyme catalytic functionality (Mendel, 2013). *Mocs1* in humans encodes two protein products via a complex alternative splicing process: a MOCS1A protein and a MOCS1AB fusion protein, both of which catalyze the first step (converting GTP (Guanosine triphosphate) into cPMP (cyclic pyranopterin monophosphate) in a complex pathway that produces the pyranopterin cofactor (Molybdenum Cofactor or “Moco”, Leimkühler, 2017). It is not clear whether the same complex alternative splicing process occurs in Drosophila, however the *Mocs1* gene structure is largely conserved (Gray and Nichols, 2000). Several critical redox enzymes use Moco, performing essential physiological and environmental functions, involving the nitrogen, sulphur and carbon cycles (Zhang and Gladyshev, 2008, Marelja *et al*. 2018). In flies, enzymes in the MOCO synthesis pathway and enzymes that require MOCO cause eye colour phenotypes that have provided early models for physiological biochemistry (Marelja **et al.* 2018).* In humans, mutations in the Moco synthesis pathway segregate with severe disease (early childhood lethal) primarily resulting from sulfite oxidase deficiency, required for cysteine catabolism (Schwarz, 2016). Sulfite oxidase is located in the intermembrane space of the mitochondrion, where electrons resulting from cysteine oxidation are passed to acceptors in the electron transport chain (Hille *et al*. 2014). The Drosophila homolog of sulfite oxidase (“*shopper*”) is required in glial cells, and modulates glutamate metabolism, required for normal neuronal excitation, with loss of function alleles displaying locomotory and behavioral defects (Otto *et al*. 2018). Moco-requiring enzymes also have “moonlighting” roles in additional biochemical processes including functions related to cellular protection and mitochondrial respiration (Gladwin *et al.* 2005). The *Mocs1* gene product is therefore feasibly situated to contribute to lifespan modulation, given the established intersection between mitochondrial homeostasis and pathways known to influence lifespan (Target of Rapamycin (TOR) inhibition, Insulin and insulin-like pathway signaling (IIS), Caloric or Dietary restriction; (Kapahi *et al.* 2004, Skorupa *et al.* 2008, Slack *et al.* 2011).

What explains the sex-specific results for both RpRNAi and MOCS1 experiments? A recent study provides suggestive evidence that loss of function in the Drosophila *Mocs1* gene may play a role in regulating male aggression (Ramin *et al.* 2019), which may affect lifespan in segregated males (as per our experimental design). There are also sex-specific responses in flies to dietary Molybdate, where treatment with low concentrations enhanced antioxidant activity whereas high concentrations were detrimental, and males were more sensitive to these effects than females (Perkhulyn *et al.* 2017). Given that aging involves a balance between reproduction vs. somatic maintenance, and the unique and costly metabolic requirements for making eggs, it is perhaps not surprising that sex-specific differences are often observed in both stress resistance (females having higher antioxidant potential) and aging (Tower 2015, Perkhulyn *et al.* 2017). Also, mitochondria are maternally inherited, thus more likely to be optimized in females for lifespan and stress resistance by natural selection (sexual antagonism Tower 2006). Related to sex-specificity, our observation that lifespan extension was only observed (in females) when the drivers were maternally inherited suggests a maternal effect, supported by similar published results concerning the UAS-GAL4 system (Slade and Slaveley 2015). One culprit may be the arthropod bacterial parasite Wolbachia which has been shown to enhance tolerance to iron stress, which may have a knock-on effect in mitochondrial turnover (Kosmidis *et al.* 2014).

Future work to solidify *Mocs1* involvement in lifespan extension would include quantitative expression analysis (RNAseq, RTQPCR, etc.) to confirm upregulation of *Mocs1* in the context of lifespan extension, and interaction studies to fit Moco biosynthesis into established regulatory networks that contribute to the biology of aging. PCR and antibiotic treatments might resolve any Wolbachia involvement. Finally, this study provides cautionary evidence when using the Harvard third chromosome TrIP lines in any experimental analysis of aging in Drosophila.

## Methods

For the MOCS1 experiment, isogenization was performed by ten generations of back crosses to an isogenized white eyed-line (*w^1118^* BL5905 see Reagents below) by following the red eye color reporter (*w^+^*) located on both the driver and responder transgenes. The isogenized driver was tested for functionality by crossing to a GFP responder and examining the progeny by fluorescence microscopy. The presence of the responder transgene in the isogenized stock was tested for by PCR. (Note isogenizing was not possible for the RpRNAi TrIP lines because the transgene reporters were not easily distinguishable by eye). Lifespans were measured using established protocols (Linford *et al.* 2013). Typically, 2-3 replicate vials (approximately 40-60 flies of a given sex) were established for each driver/responder combination. A standard cornmeal-yeast-agar food recipe was employed, albeit with rather low sucrose as is habitual in our laboratory (1% where 5-10% is more usual – this means the flies are somewhat calorically restricted (CR), so any lifespan extension is therefore likely additive to CR rather than epistatic). Flies were first collected from uncrowded bottle conditions. Newly eclosed males and females of each relevant genotype were allowed to mate for 48 hours before lifespan data were collected. Flies were transferred to fresh media every 1-2 days, at which time dead flies were removed and recorded using the dLife system developed in the Pletcher Laboratory (Linford *et al.* 2013). Constant temperature (22-25°C as measured daily in the lab) and humidity (60% approximate, based on facilities management, no major swings) conditions were maintained, with a 12:12 hour light:dark cycle. Lifespan comparisons between different genotype survivorship curves were carried out using the statistical package R within dLife (Linford *et al.* 2013). P-values were obtained using the log-rank test.

## Reagents

**Table:** 

Fly strain name	Genotype	Description	Bloomington Drosophila Stock center #	Reference
ELAV GAL4 Driver	w[*]; P{w[+mC]=GAL4-elav.L}CG16779[3]	Pan-neuronal driver	8760	Sink *et al*. 2001
LSP2 GAL4 Driver	*y[1] w[1118]; P{w[+mC]=Lsp2-GAL4.H}3*	Fat body driver	6357	Cherbas *et al*. 2003
RpS6 RNAi Responder	y[1] sc[*] v[1] sev[21]; P{y[+t7.7]v[+t1.8]=TRiP.HMS00413}attP2	GAL4 inducible responder cloned upstream of the Mocs1 locus; targets RpS6 with RNAi	32418	Zirin *et al.* 2020
RpS27 RNAi Responder	y[1] sc[*] v[1] sev[21]; P{y[+t7.7] v[+t1.8]=TRiP.HMS01581}attP2	GAL4 inducible responder cloned upstream of the Mocs1 locus; targets RpS27 with RNAi	36692	Zirin *et al.* 2020
RpL10 RNAi Responder	*y[1] v[1]; P{y[+t7.7] v[+t1.8]=TRiP.JF02520}attP2*	GAL4 inducible responder cloned upstream of the Mocs1 locus; targets RpL10 with RNAi (only used with LSP2 driver)	29356	Zirin *et al.* 2020
TrIP line background	*y[1] v[1]; P{y[+t7.7]=CaryP}attP2*	Att-P2 “landing site” background for TrIP lines	36303	Zirin *et al.* 2020
TrIP RNAi negative control line	y[1] sc[*] v[1] sev[21]; P{y[+t7.7] v[+t1.8]=VALIUM20-GAL4.1}attP2	GAL4 inducible responder cloned upstream of the Mocs1 locus; targets GAL4	35784	Zirin *et al.* 2020
Isogenic control	*w[1118]*	*w* (white eye mutant) line isogenic for chromosomes 1,2 and 3	5905	No publication, Flybase ref: RRID:BDSC_5905
Mocs1 enhancer trap	*y[1] w[67c23]; P{y[+mDint2] w[+mC]=EPgy2}EY00759*	GAL4 inducible enhancer trap in same location as TrIP landing site (stock # 36303) upstream of *Mocs1*	19808	Bellen *et al.* 2004
